# Identification and Characterization of a Novel Protein ASP-3 Purified from *Arca subcrenata* and Its Antitumor Mechanism

**DOI:** 10.3390/md17090528

**Published:** 2019-09-09

**Authors:** Zhongyi Guo, Hui Shi, Chunlei Li, Yuanyuan Luo, Sixue Bi, Rongmin Yu, Haoran Wang, Wanying Liu, Jianhua Zhu, Weijuan Huang, Liyan Song

**Affiliations:** 1 Department of Pharmacology, College of Pharmacy, Jinan University, Guangzhou 510632, China (Z.G.) (S.B.) (W.H.); 2 Biotechnological Institute of Chinese Materia Medica, Jinan University, Guangzhou 510632, China (H.S.) (C.L.) (Y.L.) (R.Y.) (W.L.); 3 Center for experimental technology, College of Pharmacy, Jinan University, Guangzhou 510632, China; 4 Broad Institute of MIT and Harvard, 75 Ames Street, Cambridge, MA 02142, USA

**Keywords:** *Arca subcrenata* protein, structural characterization, antitumor mechanism

## Abstract

Diverse bioactive substances derived from marine organisms have been attracting growing attention. Besides small molecules and polypeptides, numerous studies have shown that marine proteins also exhibit antitumor activities. Small anticancer proteins can be expressed in vivo by viral vectors to exert local and long-term anticancer effects. Herein, we purified and characterized a novel protein (ASP-3) with unique antitumor activity from *Arca subcrenata* Lischke. The ASP-3 contains 179 amino acids with a molecular weight of 20.6 kDa. The spectral characterization of ASP-3 was elucidated using Fourier Transform infrared spectroscopy (FTIR) and Circular Dichroism (CD) spectroscopy. Being identified as a sarcoplasmic calcium-binding protein, ASP-3 exhibited strong inhibitory effects on the proliferation of Human hepatocellular carcinoma (HepG2) cells with an IC_50_ value of 171.18 ± 18.59 μg/mL, measured by 3-(4,5-dimethylthiazol-2-yl)-2,5-diphenyltetrazolium bromide (MTT) assay. The RNA-seq analysis showed that ASP-3 regulated the vascular endothelial growth factor receptor (VEGFR) signaling pathway in HepG2 cells. Immunofluorescence results indicated that ASP-3 effectively reduced VEGFR2 phosphorylation in HepG2 cells and affected the downstream components of VEGF signaling pathways. The surface plasmon resonance (SPR) analysis further demonstrated that ASP-3 direct interacted with VEGFR2. More importantly, the therapeutic potential of ASP-3 as an anti-angiogenesis agent was further confirmed by an in vitro model using VEGF-induced tube formation assay of human umbilical vein endothelial cells (HUVECs), as well as an in vivo model using transgenic zebrafish model. Taken together, the ASP-3 provides a good framework for the development of even more potent anticancer proteins and provides important weapon for cancer treatment using novel approaches such as gene therapy.

## 1. Introduction

Malignant tumors pose a serious threat to human health and result in high global morbidity and mortality. According to a report, in 2015, the number of new malignant tumor cases in China was about 42.92 million, and the number of deaths caused by this kind of tumor was about 2.814 million [1]. Globally, cancer is still a critical health threat that remains to be solved. Therefore, it is extremely urgent to find safe and effective antitumor drugs [2]. Surgical resection combined with radiotherapy and chemotherapy can reduce cancer mortality, but surgical treatment may effectively remove only macroscopic tumors. Many small molecule antitumor drugs were developed over the past decades. However, while killing tumour cells, chemical drugs often have severe toxicity on normal cells, which impairs patient immunity, increases recurrence rates, and reduces survival rates. Meanwhile, most types of cancers have the potential for high invasiveness and subsequent recurrence and metastasis, which is especially common in liver cancer. Liver cancer is recognized as the fourth leading cause of cancer mortality in the world [3]. Although several signaling pathways have been consistently found dysregulated in hepatocellular carcinoma (HCC) (e.g., WNT-β-catenin, PI3K/AKT/MTOR, RAS/MAPK, IGF, HGF/MET, VEGF, EGFR, and PDGF) [4], the incomplete understanding of the molecular mechanism of HCC contributes poor overall prognosis in HCC patients [5]. Several studies have focused on the vascular endothelial growth factor (VEGF)/vascular endothelial growth factor receptor 2 (VEGFR2) axis, which can trigger multiple downstream signaling pathways [6], causing tumor cell proliferation, migration, and angiogenesis. Both VEGF and VEGFR are expressed in a variety of HCC cell lines, including HepG2, BEL-7402, SMMC-7721, Huh7, and MHCC-97H. Therefore, the discovery of new antitumor drugs with high selectivity and low toxic effects from natural products has become a key area of investment by researchers worldwide. The VEGF/VEGFR axis has also become an ideal target for the development of molecularly targeted agents.

Marine organisms are a promising pool of unexploited weaponry, especially proteins which are of an ample structural diversity and have displayed various biological activities. Although foreign protein can hardly be developed into an ideal drug via oral intake or injection of its protein form, it can be combined with the viral vector or mRNA technology to reach the target to exert a local and long-term effects, without severe off-target toxicity. Different from small molecules, proteins can also be quickly evolved and optimized in vitro to exhibit various characterizations, just like how GFP proteins were optimized [7]. 

Marine proteins are mainly isolated from marine organisms such as sponges [8], sea squirts [9], mollusks [10], algae [11], marine fungi [12], and marine strain [13]. A large number of investigations have shown that marine proteins exert good antitumor effects. For instance, some constituents discovered from the sea hare *Dolabella auricularia*, e.g., dolastatins, contain unique peptides, which have shown great potential in industrial usages, especially for developing novel and efficient anticancer drugs [14]. Conotoxin is another class of antitumor polypeptides that act on ion channels and neuroreceptors. It has been found that some conotoxins such as κ-conotoxins-PVIIA can act on potassium channel proteins, encoded by the *HERG* gene, on tumor cell surface in order to inhibit tumor cells proliferation [15]. It has been reported that the cyclic peptide aplidine, isolated from marine tunicate *Aplidium alicans*, blocked the secretion of VEGF from MOLT-4 cells [16]. In recent years, several research groups have studied the antitumor proteins from marine organisms, e.g., blue mussel [17], oyster [18], *Meretrix meretrix* [19], and *Donax variabilis* [20].

*Arca subcrenata* Lischke is a marine organism belonging to the Arcidae family, mainly found in the waters surrounding in China, South Korea, Japan, and Southeast Asia. The organism is a famous seafood due to the fact of its flavor and high nutritional value and also considered as a Chinese medicinal ingredient of benefit to digestion and cancer management [21]. Chinese material medica wa leng zi (Concha Arcae) is derived from the shell of *A. subcrenata* [22]. Earlier in 2007, a water-soluble polysaccharide isolated from *A. subcrenata* Lischke was reported to markedly stimulate mouse spleen lymphocyte proliferation [23]. Also, a polypeptide fraction from *A. subcrenata* was proved to be capable of downregulating immune-related gene transcription or inhibiting the production of inflammatory cytokines including IL-6, IL-8, and TNF-*α* [24]. In recent years, studies on the anti-tumor effects of *A. subcrenata* become increasingly popular. In 2012, a report showed that a polypeptide fraction from *A. subcrenata* displayed good antitumor activity in HeLa and HT-29 cell lines with low toxicity in vitro and in vivo [25]. In 2015, more in-depth reports showed that the polypeptide fraction from *A. subcrenata* exerted antitumor activation in inhibition of MAPKs signaling [26]. So far, plenty of research has focused on the chemical characteristics or effects of *Arca* species, but there are few reports on the relationship between their chemical structure and mechanism, which is vital for potential drugs.

The present study was one part of our effort to find antitumor proteins from the *Arca* species. In this work, we isolated and purified a novel protein named ASP-3 from *A. subcrenata*, and its complete amino acid sequence and physicochemical properties were determined. The antitumor mechanism and potential binding target of ASP-3 were also investigated for the first time.

## 2. Results

### 2.1. Isolation, Purification, and Identification of ASP-3

**Figure 1:**
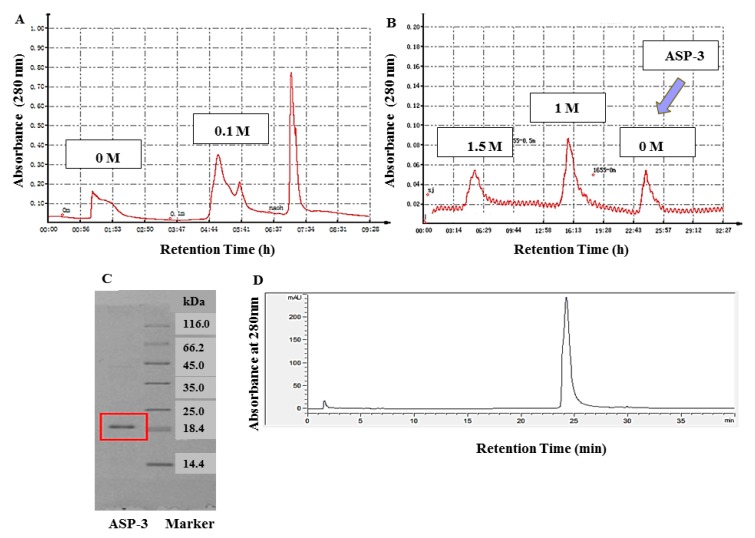
Isolation, purity, and molecular mass analysis of ASP-3. (**A**) Separation of proteins by diethyl-aminoethanol (DEAE)-sepharose fast flow anion exchange chromatography; (**B**) purification of the protein fraction by phenyl sepharose CL-4B hydrophobic chromatography (the blue arrow); (**C**) molecular mass analysis of ASP-3 by SDS-PAGE (the red box); and (**D**) RP-HPLC profile of ASP-3.

We used ion-exchange chromatography and hydrophobic chromatography to purify the proteins after the protein fractions were separated by ammonium sulfate precipitation (Figure 1A,B). The freeze-dried powder was obtained and named ASP-3. The purity was analyzed by sodium dodecyl sulphate-polyacrylamide gel electrophoresis (SDS-PAGE) and reverse-phase high-performance liquid chromatography (RP-HPLC) (Agilent, Foster City, CA, USA). The protein content of ASP-3 was determined to be 96.625% (w/w) by the Bradford method, and its molecular weight was about 20 kDa (Figure 1C,D).

As shown in Table 1, Table 2 and Table 3, a large number of unique peptides were homologous to cds.c115914_g1_i1, a 20.6 kDa protein derived from the transcriptome of *A. subcrenata*. By applying nano ESI MS/MS sequencing analysis and in-gel digestion, we obtained the complete amino acid sequence of ASP-3 from the transcriptome unigene analysis. The full-length amino acid sequence of ASP-3 is: MDYLTSKWKLWFHSLDVNHDGKISFEDVEESRNKFTDLHKLLGDKASGVKVDMESWWNTYIFKASGGGELSEEMFIKSNTDRYNKDKAAFLNDIQKCFNMIFDVIDTNKDRSIELNEFIYAFKAFGHENEGLVTKAFELLEPKEGLVPLRDIVNAWVSFVTSTDQSKPDAIKQAFDAGF. 

Next, we blasted this sequence within the NCBI database by using the default BLAST setting. The homology of 71% was shown between ASP-3 and the sarcoplasmic calcium-binding protein-like isoform X1 derived from *Crassostrea virginica* (XP_022309560.1). 

### 2.2. Spectral Characterization of ASP-3

**Figure 2:**
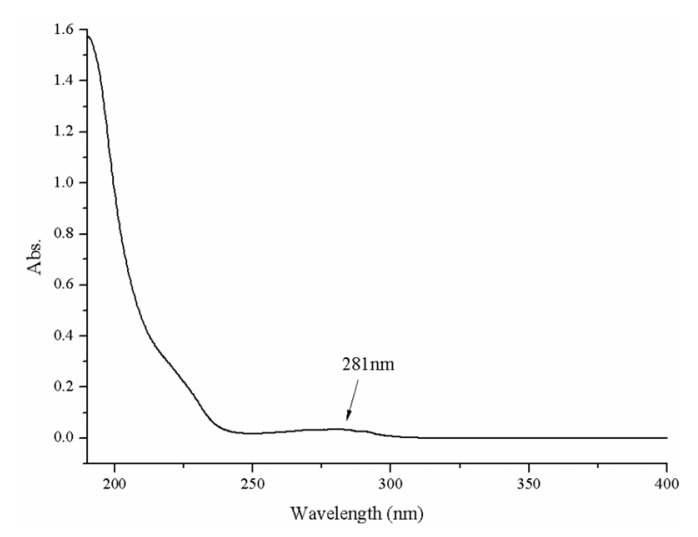
UV–Vis spectrum of ASP-3.

The 20 amino acids contained in natural proteins have no absorption peak at 230–310 nm. Only aromatic amino acids (tyrosine, phenylalanine, and tryptophan) show an absorption peak at 190–220 nm, and another absorption peak between 250 and 300 nm. An obvious absorption peak (190 nm) and a shoulder peak (281 nm) were observed in Figure 2. The two peaks showed the absorption characteristic of the protein and the aromatic amino acid residues of ASP-3, respectively.

**Figure 3:**
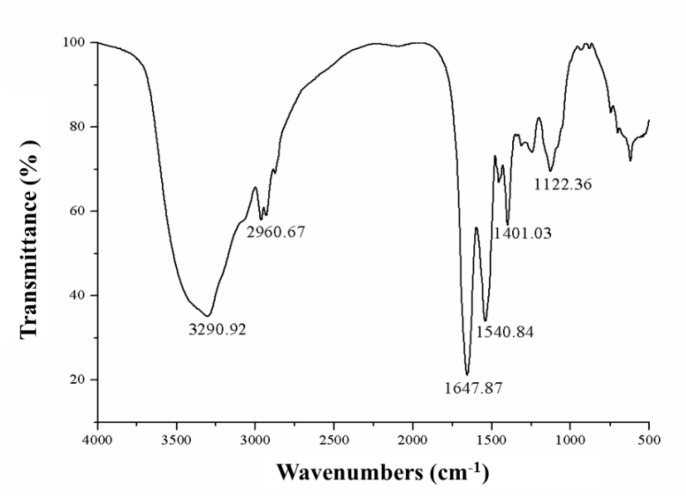
FTIR spectrum of ASP-3.

The FTIR spectroscopy has been confirmed as a useful tool for providing information on the conformational and structural dynamics of proteins. The amide bands have a relationship with the secondary structure of a protein. The widely accepted standard bands used when characterizing a protein are as follows: amide A (3000–3500 cm^−1^), amide B (2800–2990 cm^−1^), amide I (1600–1700 cm^−1^), and amide II (1300–1590 cm^−1^) [27,28,29]. As shown in Figure 3, the ordinate is the transmission percentage (T%) and the abscissa is the wavelength (unit is cm^−1^). The FTIR spectrum of ASP-3 exhibited two main bands at 1647.87 cm^−1^ (amide I) and 1540.84 cm^−1^ (amide II), respectively, which indicated that ASP-3 might be composed of α-helix structure. The other broad band was found at 3290.92 cm^−1^ (amide A), indicating that there are vibrations of hydroxyl stretching. The absorption peak at 2960.67 cm^−1^ assigned to the C–N stretching vibration was a typical amide B band peak. These data proved that ASP-3 is indeed a protein [30]. 

**Figure 4:**
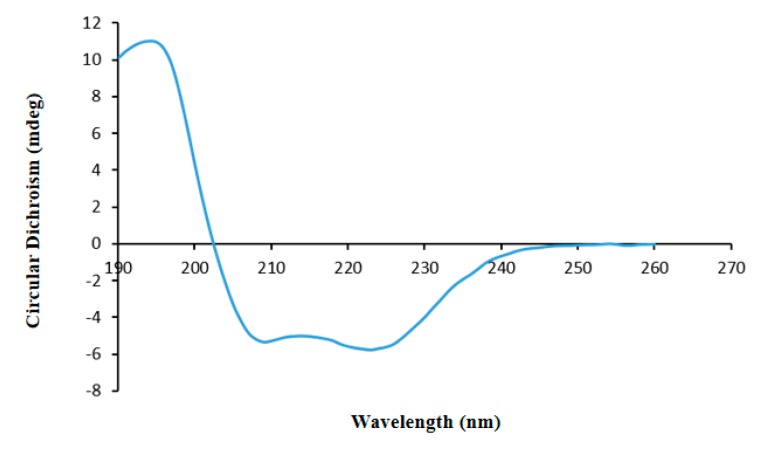
Secondary structure determination of ASP-3 by CD spectrum.

In a protein which contains α-helix structures, there are usually one positive peak (approximately 192 nm) and two negative peaks (approximately 208 and 222 nm). The CD spectrum of ASP-3 featured a 197 nm peak and two negative peaks at 208 and 222 nm (Figure 4). Thus, it is very likely that the ASP-3 protein contains α-helix structures. The secondary structure prediction programs using CDNN software 2.1 (circular dichroism analysis using neutral networks courtesy of Gerald Böhm, Martin-Luther-University Halle-Wittenberg, Germany) also generated similar results: the percentages of α-helix, β-sheet, β-turn, and random coils of the protein were 99.2%, 0.2%, 3.5% and 0.5%, respectively. Therefore, structurally speaking, ASP-3 is apparently a highly ordered and stable protein [31,32].

### 2.3. Antiproliferative Activity of ASP-3 against HepG2 Cells

**Figure 5:**
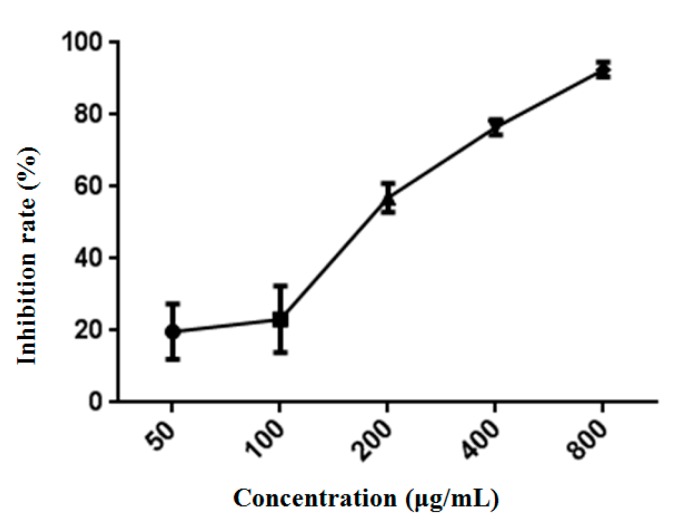
Antiproliferative activity of ASP-3 against HepG2 cells.

The MTT assay was used to detect the inhibitory effect of ASP-3 on the proliferation of HepG2 cells. As shown in Figure 5, ASP-3 inhibited the proliferation of HepG2 cells in a concentration-dependent manner, with an IC_50_ value of 171.18 ± 18.59 μg/mL.

### 2.4. Differentially Expressed Genes (DEGs) of HepG2 Cells by ASP-3 Treatment

**Figure 6:**
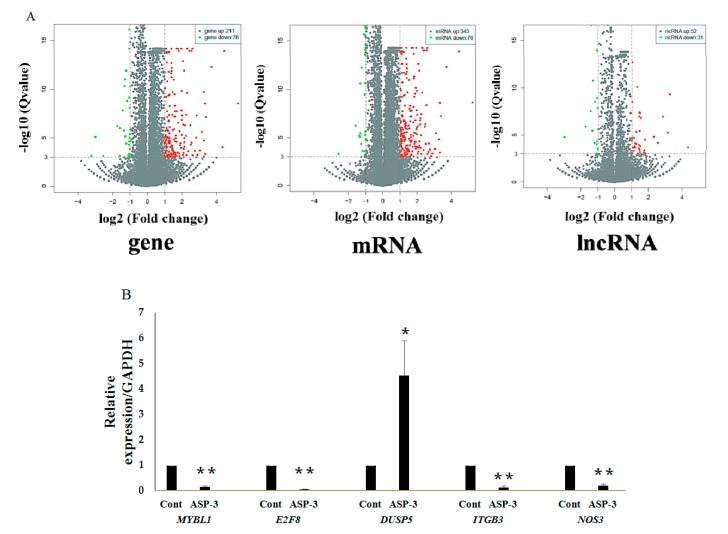
ASP-3 induced the changes in tumor-related gene expression of HepG2 cells. (**A**) Distributions of DEGs by volcano diagram; (**B**) tumor-related gene expression by qRT-PCR. * *p* < 0.05 versus control; ** *p* < 0.01 versus control.

To investigate the mechanism of antitumor action of ASP-3 against HepG2 cells, the RNA sequencing was applied. Compared with the untreated control, multiple genes of HepG2 cells with ASP-3 treatment were discovered. The differentially expressed genes between treatment and control groups were selected according to a fold change threshold and the *p*-value. The volcano diagram shows distributions of DEGs in HepG2 cells treated with ASP-3 (Figure 6A). A total of 211 genes were upregulated and 78 genes were downregulated. These results exhibited that gene expression of HepG2 cells significantly differed under ASP-3 treatment (Table 4). In total, 289 DEGs were mapped into GO terms and KEGG pathways to further understand the potential influence of ASP-3 treatment in HepG2 cells. The RNA-seq results suggested that the PI3K/MAPK/VEGF signaling pathway was the most highly regulated after ASP-3 treatment, and the five DEGs related to the PI3K/MAPK/VEGF pathway were *DUSP5*, *ITGB3*, *NOS3*, *E2F8*, and *MYBL1* (|log2FoldChange| > 1, *q*-value < 0.001) (Table 5). To confirm the differential gene expression levels of the control group and the ASP-3 group obtained by the RNA-seq assay, the gene expression levels were verified by quantitative real-time PCR (qRT-PCR) again. The results showed that the expression levels of the four genes were consistent with the RNA-seq data, indicating that the RNA-seq data was reliable. The qRT-PCR results showed that the mRNA expressions of *ITGB3*, *NOS3*, *E2F8*, and *MYBL1* were reduced and the mRNA expression of *DUSP5* increased after ASP-3 treatment (Figure 6B). 

### 2.5. Effect of ASP-3 on p-VEGFR2 in HepG2 Cells

**Figure 7:**
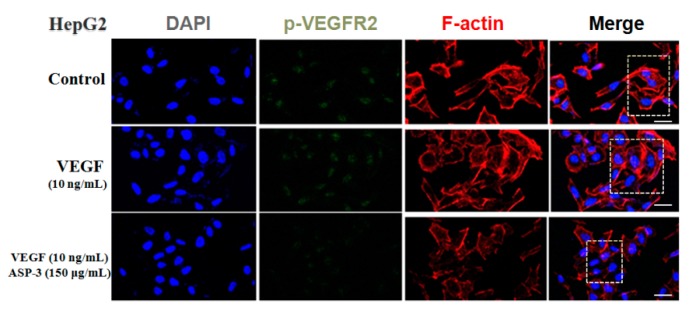
ASP-3 reduced VEGFR2 phosphorylation in HepG2 cells. Immunofluorescence staining was used to evaluate the distribution of VEGFR2 phosphorylation. They were stained with DAPI (blue), fluorescent secondary antibody of phospho-VEGFR2 VEGFR2 (green), and phalloidin (red), respectively. The immunofluorescence profile was visualized under a confocal fluoresce (scale bar: 20 µm).

Immunofluorescence was used to evaluate the effects of ASP-3 on the distribution of VEGFR2 phosphorylation using a confocal microscope. The DAPI (blue) and F-actin (red) in HepG2 cells were used to observe the profile of the cells. As shown in Figure 7, the fluorescence intensity of VEGFR2 phosphorylation (green) in the VEGF-treated group was strong compared to the control group. However, VEGFR2 phosphorylation was significantly weakened in the ASP-3 treated group. Also, the expression of F-actin was weakened, and the cytoskeleton became incomplete in the ASP-3-treated group of the cells. The cytoskeleton arrangement is one of the most important factors associated with the migration and invasion processes of tumor cells. Similarly, cell migration is one of the critical steps in angiogenesis.

### 2.6. Determination of Interaction between ASP-3 and VEGFR2

By monitoring the angular change in SPR, the kinetic binding and dissociation constant, affinity and specificity of the analyte could be automatically obtained. Biomolecular binding might also be observed. To test the capability of the interaction between ASP-3 and VEGFR2, we employed the Biacore system and immobilized ASP-3 to a CM5 chip surface, while solutions containing VEGFR2 rinsed over the chip surface. The Biacore instrument detects the binding affinity of protein–protein interactions in real time by monitoring the dynamic change in SPR while VEGFR2 interacts with ASP-3.

**Figure 8:**
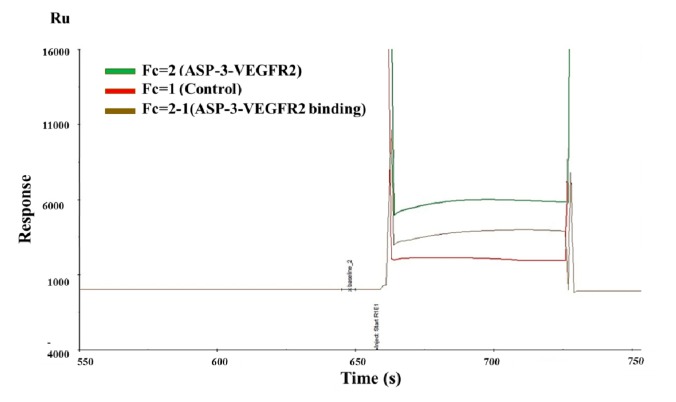
ASP-3 (100 nM) interacted with VEGFR2 based on SPR platform Biacore S200.

As shown in Figure 8, Ru was significantly higher when ASP-3–VEGFR2 was present (green line) compared to the reference channel (red line). Non-specific responses were removed from the total response, giving the time course of a specific response (orange line) [33]. The binding equilibrium dissociation constant (KD) between ASP-3 and VEGFR2 was 3.994 × 10^−8^ M. From the results, ASP-3 should be interacted with VEGFR2.

### 2.7. Molecular Docking Simulations

**Figure 9:**
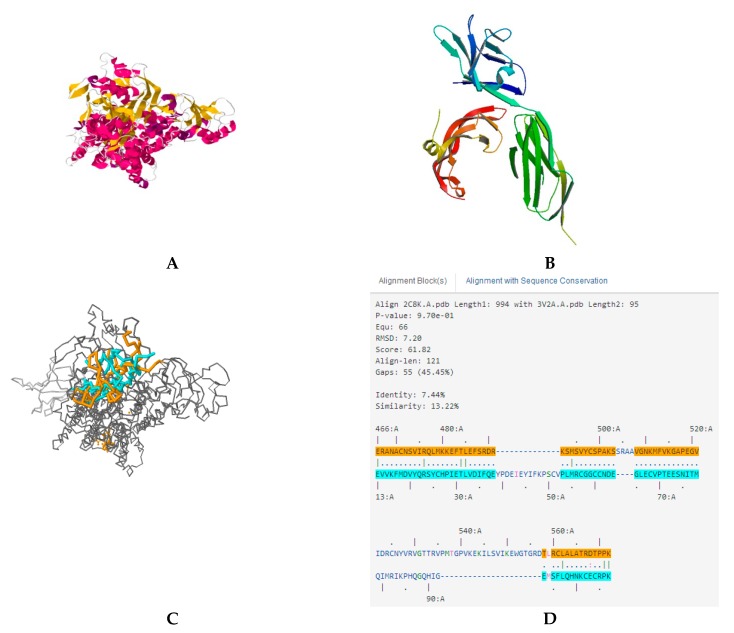
The predicted docking model and diagrams of ASP-3 and VEGF/VEGFR2. (**A**) The predicted structure of ASP-3. (**B**) The structure of VEGFA/VEGFR2. (**C**) The functions of ASP-3 and VEGFA/VEGFR2 (orange and blue). (**D**) Amino acid sequence alignment of ASP-3 and VEGFA/VEGFR2.

Molecular docking is a way to study the strength of one protein’s affinity with another protein. To further investigate the binding site between VEGFR2 and ASP-3, molecular docking was performed. The VEGFA/VEGFR2 complex structure (PDB number: 3V2A) from the Protein Data Bank (PDB) archive was used in the docking simulation. Because of the highly similar (71%) structures between ASP-3 and sarcoplasmic calcium-binding protein-like isoform X1 derived from *Crassostrea virginica* X1 (PDB number: 2C8K), X1 was used as the template to build the predicted three-dimensional structure of ASP-3. There were binding sites between ASP-3 and VEGFA/VEGFR2 complex (Figure 9) [34].

### 2.8. Effect of ASP-3 on Tube Formation Assay

**Figure 10:**
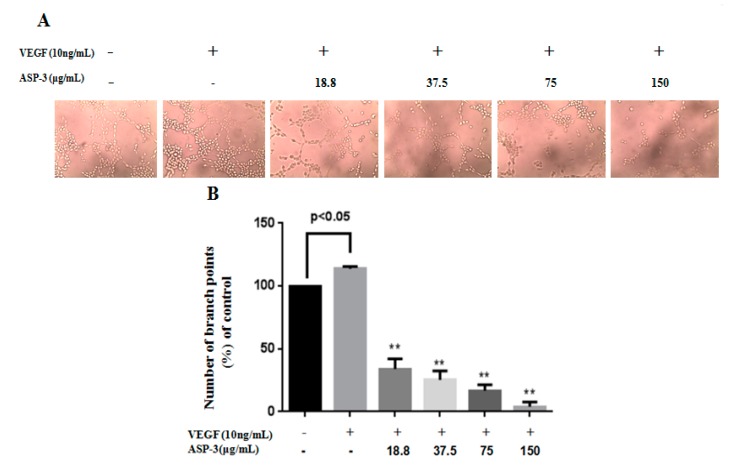
ASP-3 inhibits VEGF-induced tube formation of HUVECs in vitro. (**A**) Representative tubular structure images of HUVECs treated with different concentration of ASP-3 and VEGF (10 ng/mL, magnification 10×). (**B**) Number of branch points in HUVECs measured. *p* < 0.05 versus blank control; ** *p* < 0.01 versus VEGF-treated group.

The anti-angiogenetic effect of ASP-3 was evaluated in the tube formation assay of human umbilical vein endothelial cells (HUVECs). After 6 h of incubation, the morphological change and network structure of HUVECs placed onto the matrigel occurred in the presence of VEGF. The ASP-3 remarkably inhibited this process in a concentration-dependent manner. The ASP-3 had potent activity to inhibit the formation of endothelial tube-like structures induced by VEGF (Figure 10).

### 2.9. Inhibitory Effect of ASP-3 on Angiogenesis in a Zebrafish Model

**Figure 11:**
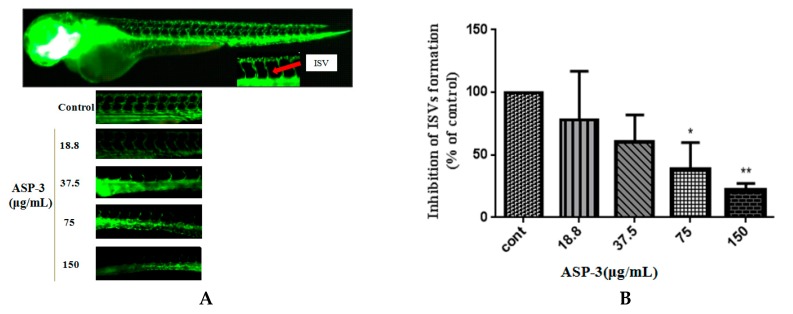
Anti-angiogenesis activity of ASP-3 in transgenic zebrafish model. (**A**) Lateral view of fli1a zebrafish embryos at 72 hpf. Live fluorescence microscopy highlights GFP expressing ISVs treated with ASP-3 in the concentration of 0–150 ug/mL (magnification: 4× and 10×). (**B**) The area of ISVs of zebrafish. (*n* = 6 for each experimental group). * *p* < 0.05; ** *p* < 0.01 versus control.

We next evaluated the anti-angiogenesis effect of ASP-3 in zebrafish embryos. The microscopic images of the transgenic zebrafish embryos showed that the intersegmental vessels (ISVs) developed a normal structure without ASP-3. By contrast, ASP-3 (18.8–150 μg/mL) hindered ISVs formation (Figure 11). From our results, we concluded that ASP-3 is likely to be associated with inhibition of ISV formation during the development of zebrafish embryos. 

## 3. Discussion

In this study, a novel protein, ASP-3, was isolated from *A. subcrenata*, which inhibited the proliferation of HepG2 cells, and its mechanism of action was firstly explored. Studies demonstrated that proteins from aquatic shelled mollusks (e.g., an oyster or cockle) or crustacean (e.g., a crab or shrimp) exhibit potential antitumor activities, making them an ideal resource for developing potential antitumor drugs. Previously, our research group reported that the novel proteins purified from *Tegillarca granosa* and *A. subcrenata* showed antitumor activity against HepG2, HT-29, and HeLa cells in vitro [35,36]. To obtain the structure–activity relationship of the compound is critical to the development and utilization of the marine organism. The amino acid sequence of the protein is the basis of the high-order structure of the protein, which determines the spatial structure and functional properties, and also an indispensable part of protein research. The structure determines the function, and the amino acid sequence has a great influence on the biological activity of the protein. Using a range of chemical characterization methods, we found that ASP-3 contained 18 lysines in the sequence of 179 amino acids, which is the largest proportion in its amino acid sequence. Some studies have reported that proteins containing more arginine or lysine residues are associated with their antitumor activities. This is probably because these two types of amino acids play a role in promoting the immune system’s function via limiting cell proliferation of the host. The amino acids are usually polar, charged, and hydrophobic. Similarly, phenylalanine and leucine, as well as other hydrophobic amino acids accounted for more in the protein sequences [37]. The FTIR spectrum results indicated that there was a certain percentage of α-helix conformation in ASP-3. Furthermore, the CD spectrum showed that ASP-3 contained 99.2% α-helix structures which confirmed that ASP-3 is a highly ordered and stable protein. Some studies have pointed out the amphiphilic peptides adopt α-helical or β-sheet structures upon binding to membranes in order to disrupt the membrane integrity or inhibit the cellular functions. The high anti-tumor activity might be due to the fact of their high percentage of α-helix folding; a peptide (polybia-MPI) was reported to induce a significant reduction of antitumor activity when the content of a-helix conformation was reduced or disrupted. Other reports have also shown that for antitumor proteins, there is a preference for the most high-activity ones which have a partial α-helix or β-sheet structure harboring an alternative sequence pattern [38,39,40]. 

Several studies have reported the applications of the transcriptome method in antitumor mechanisms of marine proteins. The RNA-seq is a widely used method for determining the abundance of transcripts and identifying new transcriptional activities through extensive detection of data. Today, many scientists use RNA-seq to study tumor function genes and their mechanisms [41], such as analysis of the potential inhibition mechanism of phycocyanin on NSCLC A549 cells by transcriptome data [42]. The RNA-seq results in our investigation showed that the expressions of ITGB3, NOS3, E2F8, and MYBL1 were reduced and the expression of DUSP5 increased after ASP-3 treatment (Figure 6B). The *E2F8* gene is a member of the atypical *E2F* (transcription factors) family, regulating cell proliferation, cell differentiation, cell cycle, as well as some fundamental cellular processes, such as DNA repair and apoptosis. It is closely related to cyclin CDK1, which could cause G2/M phase arrest in prostate cancer cells and is known to play an important role in the occurrence and development of hepatocellular carcinoma. In terms of angiogenesis, it is known that VEGF-A is activated by *E2F7* and *E2F8*, two atypical *E2Fs*. Even if there is no canonical *E2F* binding element, these two *E2Fs* still could directly bind and activate the VEGF-A promoter independently [43,44]. Both *E2F7* and *E2F8* play a critical role in angiogenesis [45]. Myeloblastosis viral oncogene homolog-like 1 (*MYBL1*) belongs to the proto-oncogene family, and this *MYB* gene has been reported as an oncogene of adenoid cystic carcinoma [46]. *MYBL1* induces tumor growth in 3T3 cells [47]. DUSP5 is a member of dual specificity phosphatases (DUSPs), belonging to the protein tyrosine phosphatases (PTPs) super family. Studies have shown that most known DUSP family members are involved in cell proliferation, differentiation, metabolism, gene transcription, ion channels, cell-to-cell communication, immune response, and tumor formation as negative regulators of MAPK [48]. In endothelial cells, two DUSP genes, *DUSP1* and *DUSP5*, were induced by VEGF [49]. *ITGB3* belongs to the integrin family, and integrin has some important functions in the occurrence and development of malignant tumors. It affects tumor metastasis directly or indirectly through mediating multiple links such as tumor and basement membrane, tumor and host–cell adhesion, signal transduction, and PI3K can transmit the invasion signal mediated by integrin [50]. Finally, NOS3 is nitric oxide (NO) synthetase, and NO plays an important role in various steps of VEGF-mediated tumor angiogenesis, such as dissolution of matrix, cell migration and proliferation, new vascular network construction, and lumen formation. Additionally, VEGF-mediated NO production has been found via eNOS upregulation by VEGFR-2 signaling [51]. The five genes above are closely related to VEGF, affecting the occurrence and development of tumors. 

VEGF is an important angiogenic factor; there are five members of VEGF, namely, VEGF-A to -D and placental growth factor (PLGF). In vivo, these proteins bind to the VEGF receptors (VEGFRs), triggering downstream cellular responses. In addition, for VEGFR, three members, namely, VEGFR-1 to -3 regulate the angiogenic function of VEGF when ligating with them. It is known that VEGF-A plays an important role in the signal transmission of the VEGFR-2-mediated tumor angiogenesis. Disturbing the tyrosine kinase VEGFR-2 signaling pathway usually results in perturbation of the angiogenesis process. The binding of VEGF to VEGFR is considered to be the most important and specific prerequisite for angiogenesis. VEGFR2 is mainly expressed in vascular endothelial cell membrane and is involved in transduction and also the core node in the whole angiogenic signal network and plays a pivotal role. Mitogen-activated protein kinase (MAPK) and phosphoinositide 3-kinase (PI3K) are the major cell growth promoting signaling pathways and play a key role in the control of cell proliferation, metabolism, and survival. These two signal pathway abnormalities are the main causes of cancer, tumor invasion, metastasis, and drug resistance. In addition, the abnormalities of these two signaling pathways occur extensively in many types of tumors and often exist simultaneously. According to the results of RNA-seq, we hypothesized that the inhibition of ASP-3 on HepG2 cells related to PI3K/MAPK/VEGF pathway, ASP-3 could be bound to VEGFR2. Results in immunofluorescence assay of HepG2 cells showed that ASP-3 reduced the phosphorylation of VEGFR2, therefore, to influence the downregulation of the MAPK and PI3K signaling pathways.

In modern drug research and development, computer software has become an important adjunct that plays a leading role in the understanding of drug and drug–receptor interactions at the supramolecular level. Using simulated docking helps to study the action between new compounds to drug targets. The docking results in our investigation indicated that ASP-3 and VEGFA/VEGFR2 complexes could be combined (Figure 9).

In this study, we also explored the effects of endothelial cells on tube formation and the angiogenic responsible for zebrafish vessel development of ASP-3. ASP-3 impaired one of the critical steps of angiogenesis, tube formation of HUVECs in a dose-dependent manner. It is known that angiogenic inhibitors may inhibit VEGFR-1 and VEGFR-2, causing ISV sprouting abnormality. Our results in the transgenic zebrafish model revealed that the inhibitory effect of ASP-3 on ISV formation might be associated with the suppression of the VEGF/VEGFR signaling pathway by interacting with VEGFR2 (Figure 11) [52,53]. 

**Figure 12:**
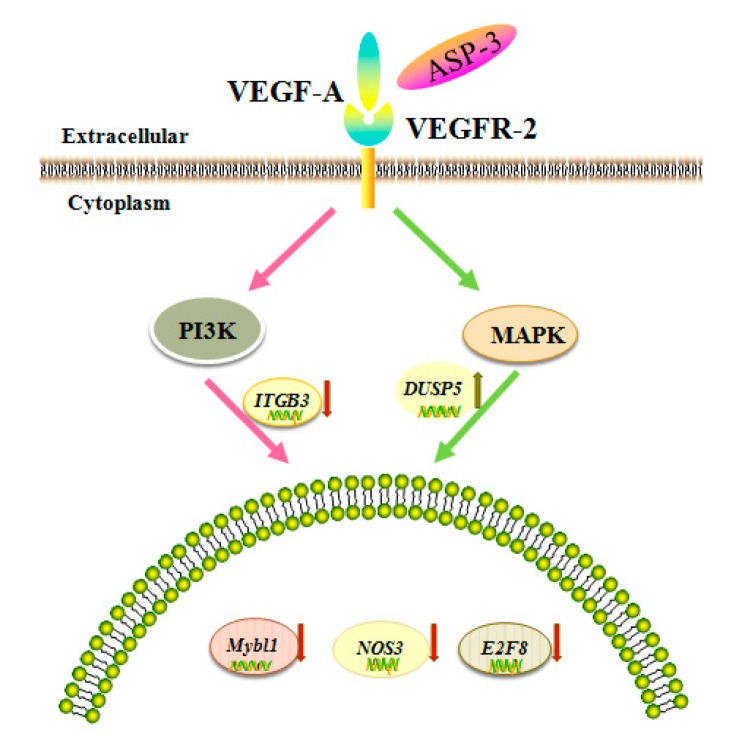
Proposed mechanism of antitumor effect of ASP-3.

The data above can help to understand that ASP-3 could neutralize to VEGFR2 and potently inhibit tumor cell proliferation and angiogenesis (Figure 12). Further work may focus on further optimization of the ASP-3 protein for anticancer properties and its application in gene therapy using mRNA or viral vectors.

## 4. Materials and Methods

### 4.1. Isolation and Purification of Protein

Ammonium sulfate precipitation is a common technique for crude protein separation. Protein solubility is closely related to salt concentrations. Subsequent chromatography could be used for further purification of the protein with high efficiency.

*A. subcrenata* materials were purchased from Huangsha seafood market in Guangzhou, China. For the protein purification, we combined ammonium sulfate fractionation with hydrophobic and anion exchange chromatography (all performed at 4 °C). The details of the procedures are described as follows. 

Approximately 200 g of *A. subcrenata* without shells were measured and rinsed by Milli-Q water. Then, the samples were homogenized by adding 600 mL phosphate buffer (pH 8.0). The ultrasonic instrument (Kunshan, China) was used to extract total proteins for 40 min. After centrifuging at 10,000× *g* for 30 min, the supernatant was considered as total protein. According to the saturated concentration of ammonium sulfate, the salting-out concentration was gradually increased, and the total protein solution was extracted three times. Finally, the protein precipitate was dissolved in 30 mM Tris-HCl (pH 8.0) buffer. To remove the remaining ammonium sulfate, we performed a dialysis to the solution. After the samples were dialyzed in distilled water and lyophilized, they were stored for further use.

First, the lyophilized protein was fully dissolved in 30 mM Tris-HCl buffer (pH 8.0). Then, the solution was loaded onto a DEAE Sepharose fast flow (GE Healthcare, Chicago, IL, USA) anion exchange column (2.5 × 40 cm). To purify the solution, the column was eluted with 0, 0.1, and 0.3 M NaCl-Tris-HCl buffers. The flow rate was set to 1 mL/min, and the protein was detected by a protein detector (Jiapeng, China). The fraction at a concentration of 0.1 M was collected at 280 nm, lyophilized, and stored for use.

In the next step, 1.5 M (NH_4_)_2_SO_4_ phosphate buffer (pH 8.0) was added to the lyophilized samples that eluted from the column. After mixing, we loaded the solution onto a Phenyl Sepharose CL-4B hydrophobic (GE Healthcare, USA) chromatography column (2.5 × 40 cm). Similarly, the column was eluted stepwise with 1.5, 1.0, and 0 M (NH_4_)_2_SO_4_ buffers. The flow rate was also set to 1 mL/min. A protein detector was used to collect proteins at 280 nm. After the last round of elution and dialysis, and the lyophilizing process, a defined protein, namely, ASP-3, was obtained.

### 4.2. Determination of Molecular Weight and Purity of Protein

Protein molecular weight and purity were determined by sodium dodecyl sulfate polyacrylamide gel electrophoresis (SDS-PAGE). The concentration of the used acrylamide and separation gel was 5% and 16%, respectively [54,55]. The molecular weight of the protein was determined in comparison to the Unstained Protein 26610 molecular marker kit (Thermo Fisher Scientific, Madison, CT, USA). The protein was visualized by staining the gel with Coomassie Brilliant Blue-250 (Thermo Fisher Scientific, Madison, CT, USA). 

Reversed-phase high-performance liquid chromatography (RP-HPLC) is featured by its high resolution and separation efficiency. The protein purity was further confirmed by using RP-HPLC (Agilent, Foster City, CA, USA). The protein was loaded onto 5 μm ZORBAX® 300SB-C8 column (4.6 × 150 mm, Agilent, Foster City, CA, USA) linked to a series 1100 HPLC system which was pre-equilibrated with 0.1% (v/v) TFA for 2 min. In the solvent, there were solvent A and solvent B, which were 0.1% (v/v) TFA in Milli-Q water and 0.1% (v/v) TFA in acetonitrile, respectively. The flow rate was set to 1 mL/min, and the linear gradient process for solvent A (40–90%) and B (90–100%) was 30 min and 10 min, respectively. Then, the protein was kept in 100% solvent B for 5 min. The detection wavelength was 280 nm and the column temperature was 30 °C [56,57].

### 4.3. In-Gel Digestion Preparation and Tandem Mass Spectrometric Identification

Tandem mass spectrometry and in-gel hydrolysis by trypsin, chymotrypsin, and rLys-C protease are standard procedures to identify the full-length amino acid sequence of proteins. These techniques, on one hand, generate robust results, and on the other hand, need less time and effort [58]. 

For in-gel digestion, we first performed a lyophilization protein, and then loaded the protein onto SDS-PAGE. The gel used was a gradient gel (5–16% polyacrylamide) and the dye was Coomassie Brilliant Blue R250. After electrophoresis, the gel containing protein was cut into small blocks and rinsed using water. DL-Dithiothreitol (DTT) (50 mM) was used to treat the blocks for 1 h at 56 °C and then to alkylate the proteins; 100 mM iodoacetamide was used to incubate the blocks for 40 min at 25 °C in dark condition. The sample was subjected to a desalting process. After being treated with a pre-made mixture, including trypsin, chymotrypsin, and rLys-C protease (E:S 1:50 m/m), at 37 °C for 20 h, the sample was heated at 56 °C for 0.5 h. In the last step, a vacuum centrifuge was first used to dry the sample, and then 20 μL of 1% (v/v) formic acid was added to solubilize the sample. The sonicated sample was loaded to a tandem mass spectrometry for multiple analyses. For the enzyme-hydrolyzed samples, their identification was done by combining reversed-phase nano-chromatography coupled to and nano-electrospray high-resolution mass spectrometry. Peptides that were desalted and hydrolyzed by the same pre-made mixture were loaded onto a 75 µm × 25 cm C18 column equipped with an EASY-nLC 1200 instrument (Thermo Fisher Scientific, Madison, CT, USA). The rate was set to 300 nL/min. In terms of the mobile phase, phrase A was 0.1% (v/v) formic acid in acetonitrile (2%) and phase B was formic acid of the same concentration in acetonitrile with a higher concentration (80%). The stepwise gradient setting was first 5–23% of B for 40 min, followed by 23–29% of B for 10 min, and then the concentration of B was set up to 100% for 6 min and maintained at 100% B for 8 min. Subsequently, eluted proteins were transferred to a nano ESI Q-Exactive mass spectrometer (Thermo, USA). High-resolution (*m*/*z* 350–1300) MS survey scans were obtained. As many as 20 ions with high intense were seeded to HCD fragmentation of the spectrum. The detailed procedure of nano ESI–MS/MS identification was described previously. The Xcalibur 4.0 software (Thermo Fisher Scientific, Madison, CT, USA) was used to obtain and analyze the data [59,60,61].

The transcriptome sequence of *A. subcrenata* was downloaded (accession number: SRR2984332) from the National Center for Biotechnology Information GenBank database (NCBI, www.ncbi.nlm.nih.gov/) Short Read Archive. Our MS/MS data of the protein was compared with the specific sequencing database of *A. subcrenata* and aligned the data with NCBI using its blast function.

### 4.4. UV–Vis Spectroscopy Determination

A Shimadzu UV-2450 model UV–vis absorption spectrophotometer (Osaka, Japan) was employed. First, we prepared a protein solution of sample (0.05 mg/mL). To refine the solution, we filtered it using a microporous membrane (0.45 μm) and the filtrate was transferred into a quartz cell (1 cm path length). Milli-Q water served as a control and the whole wavelength ranging from 190 nm to 400 nm. The flow rate was medium and the sampling interval was set to 1 nm [62].

### 4.5. Infrared Spectroscopy Analysis

FTIR spectroscopy is a well-known approach for revealing the structural information of proteins. Two amide bands, amide I and II bands, are proved to be critical for determining the secondary structure of proteins. Amide I band is mainly C–O stretch and often found at 1600–1700 cm^−1^; amide II band is usually found at 1550 cm^−1^ and the bending mode is the N–H bending mode and the stretch type is C–N. It is known that these two amide bands are closely associated with proteins’ secondary structure [63]. The helix structure of a protein makes the absorption band frequencies concentrated at approximately 1650 and 1540 cm^−1^. A FTIR spectrometer, model EQUINOX55 (Bruker, Bremen, Germany) was employed for this determination. The sample (1 mg) (pre-lyophilized) was placed in a P_2_O_5_-containing dryer. When it was completely dry, we added potassium bromide powder and compressed them into small rounded pieces. With a 2 cm^−1^ resolution, 100 scans were obtained at the range of 4000 and 400 cm^−1^. The results showed the absorption intensity by the light transmittance T% as the ordinate and the wave number (cm^−1^) as the abscissa. The ratio of the transmitted light intensity (I) to the incident light intensity (I_0_) is known as the transmittance.

### 4.6. Circular Dichroism Spectroscopy Analysis

CD spectroscopy is a commonly used detective method for studying the conformation and function relationship of organic macromolecules such as proteins and peptides. This method is surely to be accurate, simple and rapid. The CD spectroscopy offers a straightforward solution of revealing protein secondary structure. It is mainly the sum of circular dichroism in both the chromogenic group and the folded structure. The absorption of the far-UV CD spectrum at 190–260 nm is mainly used to analyze the composition ratio of the secondary structure of the protein.

Before CD analysis, the protein solution (0.02 mg/mL) was filtered through a membrane (0.02 μm). The temperature of the CD analysis was 20 °C, and the spectrometer was 101 Chirascan-plus Circular Dichroism Spectrometer made by Applied Photophysics Ltd. (Leatherhead, Surrey, UK). The quartz cells used have a 0.1 cm optical path length and a 250–190 nm scan range was selected. The CD spectrum was calibrated for the contribution of the solvent, and the data was presented ratios of specific ellipticities to wavelength. After obtaining the data, helix, sheet, turn and random coil proportions were analyzed by the CDNN software 2.1 (Courtesy of Gerald Böhm, Martin-Luther-University Halle-Wittenberg, Halle, Saale, Germany). The analysis was repeated three times.

### 4.7. Cell Culture

The HepG2 cells and HUVECs were purchased from the Type Culture Collection of the Chinese Academy of Sciences (Shanghai, China). The culture medium was RPMI-1640 medium (Gibco) containing 10% fetal bovine serum (FBS) (Gibco), 100 U/mL penicillin, and streptomycin (100 μg/mL). The cells were routinely maintained at 37 °C in humidified atmosphere with 5% CO_2_.

### 4.8. Cytotoxicity Assay

The culture medium of HepG2 cells was the same as described above, and the condition was 5% CO_2_ and 37 °C. Cancer cells at an exponentially growing stage were injected into 96 well culture plates. The density of cells to 2 × 10^3^ cells/well was controlled and incubated at 37 °C in a humidified incubator (5% CO_2_) for 24 h. The cells were subjected to treatments of sample with a series of concentrations for another 48 h. Untreated cells served as the negative control. All experiments were repeated three times and data are presented as mean ± SD.

### 4.9. RNA-Sequencing Analysis

Total RNA was isolated from the cells which were treated with ASP-3 (150 μg/mL) for 48 h. Trizol (invitrogen) method was used and the procedure was set as per the manufacturer’s instruction. An ND-1000 Nanodrop was used to determine the RNA purity. All RNA samples had an A260:A280 ratio higher than 1.8 and an A260:A230 ratio value higher than 2.0. To make sure the integrity of our RNA samples was acceptable, an Agilent 2200 Tape Station (Agilent Technologies, USA) was used to check the RINe of our samples. Only samples with a RINe higher than 7.0 was kept. In the next refining step, an Epicentre Ribo-Zero rRNA Removal Kit (Illumina, San Diego, CA, USA) was used to filter rRNAs from the total RNA. Also, the same kit was used to break the long RNA into approximately 200 bp fragments. The cDNA synthesis and adaptor ligation were done using a NEB Next Ultra Directional RNA Library Prep Kit for Illumina (New England Biolabs, Inc., Ispawich, USA). The construction of the library was completed with the Agilent 2200 Tape Station and Qubit 2.0 (Life Technologies, Carlsbad, CA, USA). All libraries were diluted to 10 pM for cluster generation in situ on the HiSeq2500 pair-end flow cell and then loaded to HiSeq3000 for sequencing (2 × 150 bp). 

We conducted qRT-PCR to verify some differentially expressed genes found in our RNA-seq results. For qRT-PCR, HepG2 cells were first treated with ASP-3 (150 μg/mL) for 48 h and then total RNA was extracted. The cDNA reverse-transcription was done using a Revert Aid First Strand cDNA Synthesis Kit (Roche, Basel, Switzerland). The qRT-PCR was performed using SYBR™ Select Master Mix (Thermo-Fisher Scientific, USA) on a Light Cycler 96 Sequence Detection system (Roche, Switzerland), and all the procedure followed the manufacturers’ instructions. The 2-ΔΔCt method was used to determine the relative gene expression and GAPDH. The ASP-3 served as reference for normalization. All primer sequences are listed in Table 6 [64,65].

### 4.10. Immunofluorescence 

The HepG2 cells (1 × 10^4^ per well) were seeded into Confocal laser (Corning, New York, NY, USA) and cultured at 37 °C in a humidified incubator with 5% CO_2_ for 24 h. ASP-3 (150 μg/mL) and VEGF (10 ng/mL) were added to the wells and incubated at 37 °C for 24 h. The cells were washed twice by PBS. Then, 4% paraformaldehyde was added and incubated for 20 min and finally adding 0.2% Triton X-100 for 5 min. Then, the cells were treated with 5% bovine serum albumin (Solarbio, Beijing, China) for 2 h. After that, p-VEGFR2 antibody was added to confocal laser overnight at 4 °C and then incubated with FITC goat anti-rabbit antibody (Invitrogen, Grand Island, NY, USA) for 2 h. Subsequently, the dishes were washed three times with PBS, incubated with DAPI and phalloidin for 5 min, respectively, and finally observed using a Leica TCS SP2 microscope (LeicaBiosystems Nussloch GmbH, Heidelberg, Germany) [66].

### 4.11. Binding Constant Determination by SPR Analysis

Biacore is a system based on surface plasmon resonance (SPR) technology to track biomolecular interactions in nature in real time. SPR is an optical phenomenon in which a metal film of about 50 nm thick is formed on the total reflection interface of the sensor chip, and the analysis process involves coupling a biomolecule or a ligand (protein, antibody, etc.) to the surface of the biosensor, and then injecting a solution containing another biomolecule (analyte) capable of interacting with the target molecule. Both of them flow through the surface of the biosensor, and the combination of biomolecules causes an increase in the surface quality of the biosensor, resulting in a change in refractive index. 

All analyses were conducted on a Biacore S200 instrument from GE Healthcare. Other experimental materials, such as CM5 sensor chips, a thiol ligand coupling kit, and running buffer HBS–EP+ 10 containing 0.1 mol/L Hepes, 1.5 mol/L NaCl, 30 mmol/L ethylenediaminetetraacetic acid (EDTA), and 0.5% p20 (v/v), were all purchased from GE Healthcare. For protein immobilization, the protein was first immobilized on CM5 sensor chips. CM5 supplemented with sodium acetate buffer (5 mM, pH 4.5, 5.0 and 5.5). Subsequently, the sensor chip surface was activated with two rounds of N-hydroxysuccinimide (NHS, 0.1 mol/L) and 1-ethyl-3-(3-dimethyl-aminopropyl)-carbodiimide (EDC, 0.4 mol/L) injection (50:50). The flow rate was set to 10 μL/min. A series of VEGFR2 solution with final concentrations of 100, 50, 25, 12.5, 6.25, and 3.125 nM was obtained by diluting VEGFR to the running buffer. Diluted VEGFR2 solutions were spread over the protein for 180 s at a flow rate of 10 μL/min. The protein samples were flushed for 200 s at a flow rate of 30 μL/min, and in the end, the regeneration of chip surface was done by adding glycine solution (10 mM, pH 2.5) for 0.5 min and sodium hydroxide solution (0.1 M) for 1 min, both at a flow rate of 30 μL/min. The protein interaction was measured by the time course of response unit, Ru(t). To remove the interreference of the non-specific response occurred on a plain surface, VEGFR2 solutions with various concentrations were poured into the surface of plain and specific chips serially. Two software, Biacore Evaluation Software 2.0 (GE Healthcare, Chicago, IL, USA) and Biacore S200 Kinetic Summary (GE Healthcare, Chicago, IL, USA), were used to summarize and interpret the data [67].

### 4.12. Molecular Docking

To illustrate the interaction with the protein and VEGF/VEGFR, docking between ASP-3 and VEGF/VEGFR was performed in the PDB online server. The structure of ASP-3 and the VEGF/VEGFR complex were obtained from PDB [68].

### 4.13. Tube Formation Assay

Matrigel Matrix (BD, Biosciences) was thawed at 4 °C overnight. The 24 well plate was coated with 250 μL Matrigel/per well and incubated at 37 °C for 30 min for polymerization. Cells (8 × 10^4^) were resuspended in 500 μL medium containing VEGF (10 ng/mL) and different concentration of ASP-3 and seeded onto the matrigel-coated plate. After 2–8 h incubation, images before and after the drug treatments were captured using a fluorescent inverted microscope [69].

### 4.14. Anti-Angiogenesis Assay in Transgenic Zebrafish Model

Since the zebrafish genome and disease signaling pathway are highly homologous to humans, the use of zebrafish to study the mechanisms and treatments of human-related diseases has become a hot research trend. Angiogenesis is thought to be a hallmark of zebrafish development, and ISV and sub-intestinal vessel (SIV) sprouting represent the development of capillaries, arteries, and veins, respectively. If VEGFR2 signaling is blocked, intersegmental sprouting angiogenesis also shows deficiency. Based on this principle, the anti-angiogenesis agent inhibiting VEGFR2 has been used to treat cancer patients. 

In this paper, transgenic male and female zebrafishes (fli-1) were housed in a controlled recirculating water system with a 14:10 h light–dark cycle at 26 °C and the pH was 7.0–7.4. One day before the experiment, the adult male and female fli-1:GFP zebrafish were mixed and placed in the mating box. The next morning light stimulated the spawning. The embryos collected within 0.5 h were placed in a Petri dish containing the embryo medium and placed in a 28.5 °C incubator to cultivate. When the embryos develop to 24 hpf, they are transferred to 96 well plates containing embryo medium, and 2 embryos are placed in each well. The prepared drug solution was sequentially added to a 96 well plate, and 6 replicate wells were set. The zebrafish were treated with ASP-3 at different concentrations. The embryos in the control group were grown in embryo medium. After 48 h of exposure to the samples, fluorescent images of the zebrafishes were captured using a Zeiss digital camera (Oberkochen, Germany). The indicators for effectively inhibiting angiogenesis in zebrafish embryos were the weakening fluorescence intensity of zebrafish embryos and the incomplete, missing, and thin expression of ISVs [70,71].

### 4.15. Statistical Analysis

The mean values were analyzed using SPSS 16.0 software (SPSS Inc., Chicago, IL, USA), the results are presented as mean ± standard deviation and GraphPad Prism 5.0 (GraphPad Software, San Diego, CA, USA) was used to the present the data. In addition, we used the *t*-test to compare differences among groups, in which *p* < 0.05 (*) or *p* < 0.01 (**) was considered to be statistically significant.

## 5. Conclusions

In conclusion, we isolated and identified the complete amino acid sequence of antitumor protein ASP-3 from *A. subcrenata*. We employed a wide range of biochemical techniques and spectral data, such as FTIR, UV-Vis absorption, CD spectroscopy, and ESI-MS/MS to understand and elucidate the physicochemical properties and structure of ASP-3. In addition, the antitumor target of ASP-3 was also evaluated. The ASP-3 exhibited direct interaction with VEGFR2 and exerted antitumor activity against HepG2 cells at least partially by inhibiting VEGF signal pathway. Overall, this study provided insights into antitumor and anti-angiogenesis efficacy of ASP-3 from *A. subcrenata*. We proposed that the marine protein ASP-3 might be further developed as a novel protein or gene-therapy based anti-VEGFR agent and provide more avenues to treating cancers.

## Figures and Tables

**Table 1:** Peptide fragments sequence analysis of ASP-3 by trypsin hydrolysis.

Sequence	^#^PG	Protein	^#^PSMs	^#^MPA	^#^MC	Theo. MH + (Da)	^#^XCS-HT	^#^CS-HT	^#^P-PEP-S-HT
ASGGGELSEEMFIK	1	1	5	cds.c115914_g1_i1	0	1470.6781	3.594579	High	0.005789
ISFEDVEESR	1	1	80	cds.c115914_g1_i1	0	1210.5586	2.922874	High	0.001299
MDYLTSKWK	1	1	1	cds.c115914_g1_i1	1	1213.5922	2.751659	High	0.02739
LWFHSLDVNHDGK	1	1	80	cds.c115914_g1_i1	0	1567.7652	4.516015	High	0.000136
AFELLEPK	1	1	1	cds.c115914_g1_i1	0	946.5244	2.033109	High	0.04643
EGLVPLR	1	1	20	cds.c115914_g1_i1	0	783.4723	2.547228	High	0.1515
AFGHENEGLVTK	1	1	72	cds.c115914_g1_i1	0	1301.6484	4.752275	High	0.0009847
LLGDKASGVK	1	1	1	cds.c115914_g1_i1	1	987.5833	2.092421	High	0.009428
ISFEDVEESRNK	1	1	22	cds.c115914_g1_i1	1	1452.6965	3.626442	High	0.003369
MDYLTSK	1	1	7	cds.c115914_g1_i1	0	899.4178	1.727565	High	0.1352
NKFTDLHK	1	1	2	cds.c115914_g1_i1	1	1002.5367	2.358591	High	0.1699

**^#^**PGs: protein groups; **^#^**PSMs: the peptide-spectrum matches; **^#^**MPAs: master protein accessions; **^#^**MCs: missed cleavages; **^#^**XCS-HT: XCorr Sequest HT; **^#^**CS-HT: confidence Sequest HT; **^#^**P-PEP-S-HT: percolator PEP Sequest HT.

**Table 2:** Peptide fragment sequence analysis of ASP-3 protein by rLys-C protease hydrolysis.

Sequence	^#^PG	Protein	^#^PSMs	^#^MPA	^#^MC	Theo. MH + (Da)	^#^XCS-HT	^#^CS-HT	^#^P-PEP-S-HT
ISFEDVEESRNK	1	1	10	cds.c115914_g1_i1	4	1452.6965	3.665950	High	0.05819
EGLVPLRD	1	1	1	cds.c115914_g1_i1	1	898.4992	2.320368	High	0.1443
GKISFEDVEESRNK	1	1	3	cds.c115914_g1_i1	5	1637.8129	2.822085	High	0.1253
ASGGGELSEEMFIK	1	1	1	cds.c115914_g1_i1	3	1470.6781	2.409946	High	0.06772
LWFHSLDVNHDGK	1	1	25	cds.c115914_g1_i1	2	1567.7652	4.727426	High	0.07214
MDYLTSKWK	1	1	1	cds.c115914_g1_i1	2	1213.5921	2.403629	High	0.07387
ASGVKVDME	1	1	7	cds.c115914_g1_i1	2	951.4451	2.751384	High	0.09791
SRNKFTDLHK	1	1	9	cds.c115914_g1_i1	2	1245.6698	4.092405	High	0.06579
AFGHENEGLVTK	1	1	5	cds.c115914_g1_i1	2	1301.6484	4.602788	High	0.00178
DVEESRNKFTDLHK	1	1	1	cds.c115914_g1_i1	5	1717.8503	2.546801	High	0.09369
LLGDKASGVKVDME	1	1	1	cds.c115914_g1_i1	4	1477.7566	2.016416	High	0.115

**^#^**PGs: protein groups; **^#^**PSMs: the peptide-spectrum matches; **^#^**MPAs: master protein accessions; **^#^**MCs: missed cleavages; **^#^**XCS-HT: XCorr Sequest HT; **^#^**CS-HT: confidence Sequest HT; **^#^**P-PEP-S-HT: percolator PEP Sequest HT.

**Table 3:** Peptide fragments sequence analysis of ASP-3 protein by chymotrypsin hydrolysis.

Sequence	^#^PG	Protein	^#^PSMs	^#^MPA	^#^MC	Theo. MH + (Da)	^#^XCS-HT	^#^CS-HT	#P-PEP-S-HT
GHENEGLVTKAF	1	1	20	cds.c115914_g1_i1	1	1301.6484	3.934973	High	0.01547
KASGGGELSEEMF	1	1	2	cds.c115914_g1_i1	1	1357.5940	2.346765	High	0.1362
KAFGHENEGLVTKAF	1	1	68	cds.c115914_g1_i1	2	1647.8489	5.835577	High	0.0009434
MDYLTSKW	1	1	2	cds.c115914_g1_i1	2	1059.4815	2.338743	High	0.1578
IFKASGGGEL	1	1	1	cds.c115914_g1_i1	1	978.5254	2.468635	High	0.07504
VTSTDQSKPDAIKQAF	1	1	1	cds.c115914_g1_i1	0	1735.8861	1.956635	High	0.1823
EDVEESRNKF	1	1	2	cds.c115914_g1_i1	0	1252.5804	3.350192	High	0.01405
KAFGHENEGL	1	1	2	cds.c115914_g1_i1	1	1101.5323	3.058953	High	0.1461

**^#^**PGs: protein groups; **^#^**PSMs: the peptide-spectrum matches; **^#^**MPAs: master protein accessions; **^#^**MCs: missed cleavages; **^#^**XCS-HT: XCorr Sequest HT; **^#^**CS-HT: confidence Sequest HT; **^#^**P-PEP-S-HT: percolator PEP Sequest HT.

**Table 4:** Differential gene/mRNA/lncRNA expression.

Gene Type	Gene	mRNA	lncRNA
Sum	289	419	83
Up	211	343	52
Down	78	76	31

**Table 5:** KEGG pathway enrichment of DEGs between control and ASP-3 treatment.

Gene	Gene_Type	mRNA_Symbol	lncRNA_Symbol	Description	KEGG Pathway
*E2F8*	protein_coding	-	-	E2F transcription factor 8	-
*MYBL1*	protein_coding	-	-	MYB proto-oncogene like 1	-
*DUSP5*	protein_coding	NM_004419.3	ENSG0000027314.1	dual specificity phosphatase 5	MAPK signaling pathway
*ITGB3*	protein_coding	NM_000212.2	THC AT158	integrin subunit beta 3	PI3K-Akt signaling pathway
*NOS3*	protein_coding	NM_000603.4	ATG9B	nitric oxide synthase 3	VEGF signaling pathway

**Table 6:** Primers used in gene expression analyses.

Gene	Primer Forward (5′-3′)	Primer Reverse (5′-3′)
*MYBL1*	AGCGAATTCCATCACAGCCT	TAACACAGCGTTTGCCTCCA
*E2F8*	TTTCCTGTCAAGCGACCTCC	AAGTTTTAATATCCTGTTCGCAGAT
*DUSP5*	TCCTGAGTGTTGCGTGGATG	TGGGCCACCCTGGTCATAA
*ITGB3*	TTGGAGACACGGTGAGCTTC	TTAGGTTCAGCTTGGGCCTG
*NOS3*	AGTGGCTGGTACATGAGCAC	GGTGACTTTGGCTAGCTGGT
*GAPDH*	GACCTGACCTGCCGTCTA	AGGAGTGGGTGTCGCTGT
